# Lampreys Have a Single Gene Cluster for the Fast Skeletal Myosin Heavy Chain Gene Family

**DOI:** 10.1371/journal.pone.0085500

**Published:** 2013-12-20

**Authors:** Daisuke Ikeda, Yosuke Ono, Shigeki Hirano, Nobuhiro Kan-no, Shugo Watabe

**Affiliations:** 1 School of Marine Biosciences, Kitasato University, Sagamihara, Kanagawa, Japan; 2 Department of Aquatic Bioscience, Graduate School of Agricultural and Life Sciences, The University of Tokyo, Bunkyo, Tokyo, Japan; 3 Department of Medical Technology, School of Health Sciences, Faculty of Medicine, Niigata University, Niigata, Japan; CNRS, France

## Abstract

Muscle tissues contain the most classic sarcomeric myosin, called myosin II, which consists of 2 heavy chains (MYHs) and 4 light chains. In the case of humans (tetrapod), a total of 6 fast skeletal-type MYH genes (*MYH*s) are clustered on a single chromosome. In contrast, torafugu (teleost) contains at least 13 fast skeletal *MYH*s, which are distributed in 5 genomic regions; the *MYH*s are clustered in 3 of these regions. In the present study, the evolutionary relationship among fast skeletal *MYH*s is elucidated by comparing the *MYH*s of teleosts and tetrapods with those of cyclostome lampreys, one of two groups of extant jawless vertebrates (agnathans). We found that lampreys contain at least 3 fast skeletal *MYH*s, which are clustered in a head-to-tail manner in a single genomic region. Although there was apparent synteny in the corresponding *MYH* cluster regions between lampreys and tetrapods, phylogenetic analysis indicated that lamprey and tetrapod *MYH*s have independently duplicated and diversified. Subsequent transgenic approaches showed that the 5′-flanking sequences of Japanese lamprey fast skeletal *MYH*s function as a regulatory sequence to drive specific reporter gene expression in the fast skeletal muscle of zebrafish embryos. Although zebrafish *MYH* promoters showed apparent activity to direct reporter gene expression in myogenic cells derived from mice, promoters from Japanese lamprey *MYH*s had no activity. These results suggest that the muscle-specific regulatory mechanisms are partially conserved between teleosts and tetrapods but not between cyclostomes and tetrapods, despite the conserved synteny.

## Introduction

Myosin is a ubiquitous actin-based motor protein that drives a wide range of motile processes in eukaryotic cells. Muscle tissues contain the most classic sarcomeric myosin, called myosin II, which consists of 2 heavy chains (MYHs) and 4 light chains (reviewed in 1). MYH contains 2 loop structures, loops 1 and 2, which are located in its N-terminal half, called the motor domain or subfragment-1 (S1), at the ATP- and actin-binding sites, respectively. MYH transduces chemical energy produced by splitting ATP to mechanical energy through the C-terminal half, called a rod, which has an α-helical coiled-coil structure.

 Various types of sarcomeric MYH genes (*MYH*s) have been found in mammalian muscles. For example, 2 fast skeletal *MYH*s, called embryonic *MYH3* and perinatal *MYH8*, are expressed during pre- and postnatal development, respectively [2]. Three *MYH*s, types IIa (*MYH2*), IIb (*MYH4*) and IId/x (*MYH1*), are expressed in adult fast muscle. Extraocular MYH (MYH13) is expressed in extrinsic eye muscle. These 6 fast skeletal *MYH*s are clustered on a single chromosome, and this chromosomal organization is highly conserved among diverse mammalian species [3,4], suggesting their functional importance for temporal and spatial expression during development. However, the regulatory mechanisms involved in the gene expression and functional differences of encoded myosin molecules remain mostly unknown.

 More diverse sarcomeric *MYH*s have been found in the fast muscle of teleosts, such as the common carp *Cyprinus carpio* [5-8], zebrafish *Danio rerio* [9-12], medaka *Oryzias latipes* [13-15] and torafugu *Takifugu rubripes* [16-19], at the genomic and transcriptional levels. In addition, genomic structural and syntenic analyses have revealed that at least 13 torafugu fast skeletal *MYH*s are distributed in 5 genomic regions, 3 of which contain multiple *MYH*s forming clusters A, B and C [18] (Figure S1). These clusters are also found in the green spotted pufferfish *Tetraodon nigroviridis*, zebrafish and medaka [12,15,18], implying that such genomic arrangement is conserved in teleosts. Interestingly, human fast skeletal *MYH*s form a single cluster, but their syntenic regions in torafugu, green spotted pufferfish and medaka are duplicated, although each locus contains a single *MYH* [18].

 Based on phylogenetic, genomic structural and syntenic analyses, teleost *MYH*s in clusters A, B and C were defined as those of fast skeletal types A, B and C, respectively; non-clustered teleost *MYH*s and those in the human cluster were defined to be of the fast D type [18] (Figure S1). Moreover, mammals, including mouse [3], dog [20] and the amphibian *Xenopus tropicalis* [18] (tetrapod) have a single syntenic region similar to that of humans but not similar to that of teleosts. However, the evolutionary relationship of fast skeletal *MYH*s among vertebrates and the functional significance of their cluster formation have remained unknown.

 Lampreys and hagfishes are two groups of extant jawless vertebrates (agnathans). These vertebrates belong to a monophyletic group of cyclostomes and are thought to have diversified from a common ancestor shared with gnathostomes (jawed vertebrates) 535-462 million years ago [21]. Due to their unique phylogenetic history among vertebrates, evolutionary and developmental studies have been carried out extensively on lampreys (reviewed in 21,22). Two fast skeletal *MYH*s (*LjMyHC1* and *LjMyHC2*) and 2 non-muscle *MYH*s (*LjMyHC3* and *LjMyHC4*) have been identified from an embryonic cDNA library of Japanese lamprey *Lethenteron camtschaticum* (renamed from *Lethenteron japonicum* in the NCBI Taxonomy database) [23]. Although phylogenetic and gene expression analyses on Japanese lamprey *MYH*s were partially carried out [23], information about their gene structure and location in the genome that would be helpful in increasing our knowledge about the evolutionary relationship of vertebrate *MYH*s is not available.

 In the present study, to elucidate the evolutionary relationship among vertebrate fast skeletal *MYH*s, we compared those from teleosts and tetrapods with their lamprey counterparts. We found that lampreys possess at least 3 fast skeletal *MYH*s, which are clustered on a single chromosome in a head-to-tail manner as in tetrapods. Subsequent transgenic and transfection studies on the function of the 5′-flanking regions of Japanese lamprey fast skeletal *MYH*s demonstrated that these genes have the same transcriptional regulation as those of teleost *MYH*s but different transcriptional regulation from those of tetrapod fast skeletal *MYH*s.

## Materials and Methods

### Ethics Statement

 Zebrafish specimens were maintained under standard husbandry conditions at the School of Marine Biosciences, Kitasato University, and animal experiments were approved by the Animal Experimentation Committee of the School of Marine Biosciences, Kitasato University. The permit number for this study is 2231.

### Experimental Fish

 Mature specimens of the Japanese lamprey, *Lethenteron camtschaticum*, which were collected in a tributary of the Miomote River, Niigata Prefecture, Japan, during the breeding season in early June, were purchased from local fishermen. Fertilized eggs prepared from Japanese lamprey were incubated in freshwater at 10°C for embryonic development. Embryonic staging was based on the report by Tahara [24]. Zebrafish specimens were used for an *in vivo* promoter assay in which their embryos were incubated at 26°C.

### Analysis of a Contiguous Genomic Region Containing Fast Skeletal *MYH*s from the Sea Lamprey Genome

The sea lamprey *Petromyzon marinus* genome database (Petromyzon_marinus-3.0) was screened with the full-length cDNA sequence of a fast skeletal *MYH* from Japanese lamprey [23] (AB126173) using the BLASTn program. A BLAST search was carried out on the genomic database using the Ensembl Genome Browser (http://www.ensembl.org/index.html). From various positive hits, we chose 4 supercontigs, which were found to contain partial sequences of *MYH*s. The exon-intron structures of these *MYH*s were predicted by manual inspection. The recently released sea lamprey genome database (Petromyzon_marinus-7.0) was also used to verify the results obtained with the Petromyzon_marinus-3.0 database.

 Coding exons of torafugu, *Takifugu rubripes*, were predicted by manual inspection from the genomic sequence of scaffold_139 (database version FUGU4; http://www.ensembl.org/index.html).

 Moreover, to compare the genomic organization of the *MYH*s with that of other chordate lineages, we also analyzed the genome databases of the amphioxus *Branchiostoma floridae* (database version Brafl1; http://genome.jgi-psf.org/Brafl1/Brafl1.home.html) and the tunicate *Ciona savignyi* (database version CSAV 2.0; http://www.ensembl.org/Ciona_savignyi/Info/Index).

### Shotgun Sequencing of the *MYH* Cluster Region in Japanese Lamprey

 DNA was extracted from the muscle of an individual of Japanese lamprey using a conventional phenol/chloroform method. The genomic region containing an *MYH* cluster in Japanese lamprey was divided into 14 parts by using the long PCR method. Primers (Table S1) were designed according to the sequences obtained from the sea lamprey genome database and cDNA sequences of Japanese lamprey *MYH*s [23] (AB126173 and AB126174). PCR was carried out with PrimeSTAR GXL DNA Polymerase (Takara, Otsu, Japan) at a reaction volume of 50 μl according to the manufacturer's instructions. To construct a shotgun library, PCR products were cut into short fragments with Nebulizers (Life Technologies, Gaithersburg, MD), and the fragments were cloned into pUC118 plasmid vector using a blunt end ligation kit (Takara). Randomly selected shotgun clones in each library were sequenced using a BigDye Terminator v3.1 Cycle Sequencing Kit (Life Technologies) and ABI PRISM 3730 and 3130 xl (Life Technologies). Sequences were assembled by using the Phred/Phrap/Consed [25–27] sequence analysis software package. The determined sequences were processed using Phred, trimmed for quality, screened for vector sequences and assembled using Phrap. Quality scores were assigned automatically, whereas electropherograms and assembly were viewed and verified for accuracy using Consed.

 During manuscript preparation, Mehta et al. [28] published and released the entire genome sequence of the Japanese lamprey. Therefore, to identify the *MYH* cluster and neighbor genes in the genomic sequence, we analyzed the genome database of the Japanese lamprey (http://jlampreygenome.imcb.a-star.edu.sg/).

### Expression Analysis of Japanese Lamprey *MYH*s

 Total RNAs were extracted from the muscle tissue of an adult Japanese lamprey and embryos at stages 21, 25 and 27 using ISOGEN (Nippon Gene, Tokyo, Japan). RT-PCR was performed using primers specific to Japanese lamprey *MYH*s *1*, *2* and *5* (Table S2A) as described previously [14]. The amplification of Japanese lamprey cytoplasmic actin *LjCA1* (AB060287) was carried out to confirm first-strand cDNA synthesis.

### Phylogenetic Tree Construction

 The deduced amino acid sequences of Japanese lamprey *MYH*s *1*, 2 and 5 were aligned using the multiple sequence alignment program CLUSTAL W [29]. The neighbor-joining (NJ) tree was constructed using the Poisson correction model on Mega 5 software [30]. Bootstrap resampling analysis from 1000 replicates was used to evaluate internal branches. The maximum-likelihood (ML) tree was constructed by the quartet puzzling algorithm using TREE-PUZZLE software [31] with the following parameter settings and estimation methods: the JTT substitution model, the uniform rate heterogeneity model and exact and slow parameter estimation. Reliability values for respective internal branches were expressed as a percentage of how often the corresponding cluster was found among the 1000 intermediate trees. Lc MYHs from the Japanese lamprey *L. camtschaticum* (deduced from the genomic sequence, AB720829) were compared with Hs MYH1 (adult fast IId/x, AAD29951), Hs MYH2 (adult fast IIa, AAD29950), Hs MYH8 (perinatal fast, NP_002463), Hs MYH4 (adult fast IIb, AAD29949), Hs MYH3 (embryonic fast, NP_002461), Hs MYH13 (extraocular fast, AAD29948), Hs MYH6 (alpha cardiac, NP_002462), Hs MYH7 (beta cardiac, NP_000248) and Hs MYH14/7B (slow A, NP_065935) from human, *Homo sapiens*, and Ai MYH (striated muscle, CAA39247) from the bay scallop, *Argopecten irradians*. MYH sequences of the torafugu, *Takifugu rubripes* (Tr), were cited from our previous data [16-19].

### Preparation of GFP and DsRed Constructs and Microinjection

 Sequences in the 5′-flanking region of Japanese lamprey *MYH*s were amplified from genomic DNA by PCR and were cloned into the promoter-less phrGFP vector (Stratagene, La Jolla, CA) or pDsRed Express-1 vector (Clontech, Mountain View, CA). For the insertion of DNA fragments into the vector, additional sequences recognized by restriction enzymes (CGCGGATCC for BamHI or ATGCGCCCGCGG for SacII) were added at the 5′ end of the primers (primer sequences are listed in Table S2B), and the insertion of DNA fragments was confirmed by PCR and sequencing. To linearize and remove plasmid vector sequences that did not require reporter gene expression in zebrafish embryos, such as pUCori and antibiotics resistant-genes, PCR was carried out with PrimeSTAR GXL DNA Polymerase (Takara) at a reaction volume of 50 μl (primer sequences are described in Table S2C). PCR products were purified with a QIAquick PCR Purification Kit (Qiagen, Valencia, CA) and dissolved in sterile distilled water.

 For microinjection, the constructs were diluted to 50 ng/μl with sterile distilled water containing 0.025% phenol red and introduced into the fertilized eggs of zebrafish at the one- or two-cell stage. The fluorescence derived from transgenes in embryos was observed with a SZX 12 stereo-microscope (Olympus, Tokyo, Japan).

### Immunohistochemistry

 Zebrafish embryos were fixed overnight at 4°C with 4% paraformaldehyde in Tris-buffered saline (25 mM Tris, 137 mM NaCl, 2.7 mM KCl, pH 7.4) containing 0.1% Tween 20 (TBSTw). Fixed embryos were washed with TBSTw, and blocking was performed using 2.0% skim milk in TBSTw. Transverse sections were prepared at a thickness of 20 μm using an HM 505N cryostat (Microm Laborgeräte GmbH, Walldorf, Germany) before the first immunoreaction. As an initial antibody, anti-GFP polyclonal antibody 598 (MBL, Nagoya, Japan) was used at a dilution of 1:2500 in the blocking solution and in F59 supplied by Developmental Studies Hybridoma Bank (Iowa City, IA) at 1:20. Immunoreaction with the first antibody was performed overnight at 4°C. After incubation, the embryos were washed with TBSTw and labeled for 3 h at room temperature with the secondary antibodies anti-mouse IgG DyLight 488 and anti-rabbit IgG DyLight 549 (KPL, Gaithersburg, MD) at a dilution of 1:250 for F59 and anti-GFP, respectively. Stained sections were viewed with a LSM 510 Meta confocal microscope (Carl Zeiss, Jena, Germany).

### Preparation of Luciferase Constructs, Cell Culture and Transfections

 Sequences in the 5′-flanking region of Japanese lamprey, zebrafish and mouse *MYH*s were amplified from genomic DNA by PCR and were inserted into the promoter-less pGL3-Basic plasmid vector (Promega, Madison, WI) with the In-Fusion HD Cloning Kit (Clontech) (primer sequences are listed in Table S2D). Cell cultures and transfections were performed as described by Meissner et al. [32] with some modifications. Mouse C2C12 myoblasts were provided by the RIKEN BRC through the National Bio-Resource Project of the Ministry of Education, Culture, Sports, Science and Technology, Japan. The cells were cultured on gelatinized culture plates in a growth medium consisting of complete Dulbecco’s modified Eagle’s medium (DMEM) with high glucose (Nacalai Tesque, Kyoto, Japan) supplemented with 10% fetal bovine serum (Biowest, Nuaillé, France), 100 U/ml penicillin, 100 μg/ml streptomycin and 2.5 μg/ml amphotericin B. C2C12 cells were transfected at 70-90% confluency with 0.5 μg of promoter DNA, 0.05 μg of pRL-TK (Promega) and 1.5 μl/μg DNA of X-tremeGENE 9 transfection reagent (Roche, Basel, Switzerland). The transfection medium was replaced after 24 h with a differentiation medium consisting of DMEM supplemented with 2% horse serum (Biowest). Myotubes were harvested after 3 or 5 days. Undifferentiated myoblasts were harvested 24 h after transfection.

### Luciferase Assays

 Cells were lysed in 100 μl of Passive Lysis Buffer (Promega). Twenty microliters of lysate was subjected to luciferase assay using the Dual-Luciferase Reporter Assay System (Promega) and a Lumat LB 9507 luminometer (Berthold Technologies, Bad Wildbad, Germany) according to the manufacturer's instructions. All experiments were performed in triplicate, and the data are presented as the mean ± SD. Student's t-test was used to determine statistical significance.

### Multiple Alignment of the 5′ Flanking Sequences of *MYH*s

 The 5′ flanking sequences of Japanese lamprey, zebrafish and mouse *MYH*s were aligned with the Shuffle-LAGAN program [33]. The alignments were compared with VISTA [34] using the Japanese lamprey *MYH1* and mouse *MYH1* (IId/x) as references with Calc Window and Min Cons Width of 20 bp, respectively. To identify putative transcription factor binding sites (TFBSs) in the identified conserved non-coding elements (CNEs), we used the MATCH program to search the TRANSFAC database (http://www.gene-regulation.com/cgi-bin/pub/programs/match/bin/match.cgi) [35], where the parameters were set to the vertebrates matrix (high quality) and the cut-off was set to minimize false negative matches (minFN).

## Results

### Analysis of a Contiguous Genomic Region Containing Fast Skeletal *MYH*s from the Sea Lamprey Genome

 We searched the sea lamprey genome database (Petromyzon_marinus-3.0) using the cDNA sequence of Japanese lamprey fast skeletal *MYH* as a probe and found 6 regions encoding the partial sequences of *MYH*s in 5 contigs (Figure S2). Although vertebrate sarcomeric *MYH*s have two 5′-untranslated exons [36], it was difficult to use these exons without sequencing their transcripts. Therefore, these exons were excluded from analysis. Moreover, contigs 6974 and 38608 were analyzed as one contig because these two partially overlap.

 At the 3′-site of contig 2034 and the 5′-site of contig 15297, we found partial sequences that could be encoded by a single *MYH* referring to Japanese lamprey *LjMyHC2* (accession #AB126174 in the DDBJ/EMBL/GenBank databases). We also found that the 3′-site of contigs 6974/38608 and the 5′-site of contig 39193 contained partial sequences that could be encoded by another *MYH* homologous to Japanese lamprey *LjMyHC1* (AB126173). At the 3′-site of contigs 15297 and the 5′-site of contig 6974/38608, we found partial *MYH* sequences that had not been reported before. For convenience in the present study, we renamed *LjMyHC1* and *LjMyHC2* as Japanese lamprey *MYH1* and *MYH2*, respectively. Because Kusakabe et al. [23] reported 2 non-muscle *MYH*s (*LjMyHC3* and *LjMyHC4*) in the Japanese lamprey, we named this newly identified *MYH* as *MYH5*. As summarized in Figure S2, sea lamprey fast skeletal *MYH*s are strongly suggested to be clustered on a single chromosome in a head-to-tail manner as in their mammalian counterparts.

 In addition, we searched the recently released sea lamprey genome database (Petromyzon_marinus-7.0) to verify our results described above. Although we found no fast skeletal *MYH* other than those obtained with the Petromyzon_marinus-3.0 database, scaffold GL477325 contained not only 2 *MYH*s (*MYH2* and *MYH5*) but also a growth arrest-specific protein 7 gene (*GAS7*) connected to the 3′-site of the *MYH* cluster (Figure S2). Scaffold GL47325 contains a region with a long sequence gap, and contigs 6974/38608 and 39193 are expected to be located in this gap region (Figure S2). Although the length of the intergenic region between *MYH1* and *GAS7* is estimated to be 20 kb, the presence of other genes between *MYH1* and *GAS7* remains a possibility. *GAS7* is also located next to the 3′-site of the fast skeletal *MYH* cluster of tetrapods such as humans and mice and the 3′-site of non-clustered *MYH*s of torafugu, green spotted pufferfish and medaka [18] (Figure S1). 

### Analysis of a Contiguous Genomic Region Containing Sarcomeric *MYH*s from the Amphioxus and Tunicate Genomes

 We searched the *Branchiostoma floridae* and *Ciona savignyi* genome databases using the cDNA sequence of Japanese lamprey fast skeletal *MYH* as a probe and found that both *B. floridae* and *C. savignyi* possessed *MYH* clusters (Figure S1). As shown in Figure S1, at least 12 *MYH*s are clustered in scaffold_192 of the *B. floridae* genome database. Furthermore, *C. savignyi MYH*s are distributed in 2 genomic regions, both of which contain at least 2 *MYH*s (Figure S1). However, there is no synteny among vertebrate, amphioxus and tunicate *MYH* cluster regions (Figure S1).

### Exon-Intron Organization of Japanese Lamprey *MYH*s

**Figure 1:**
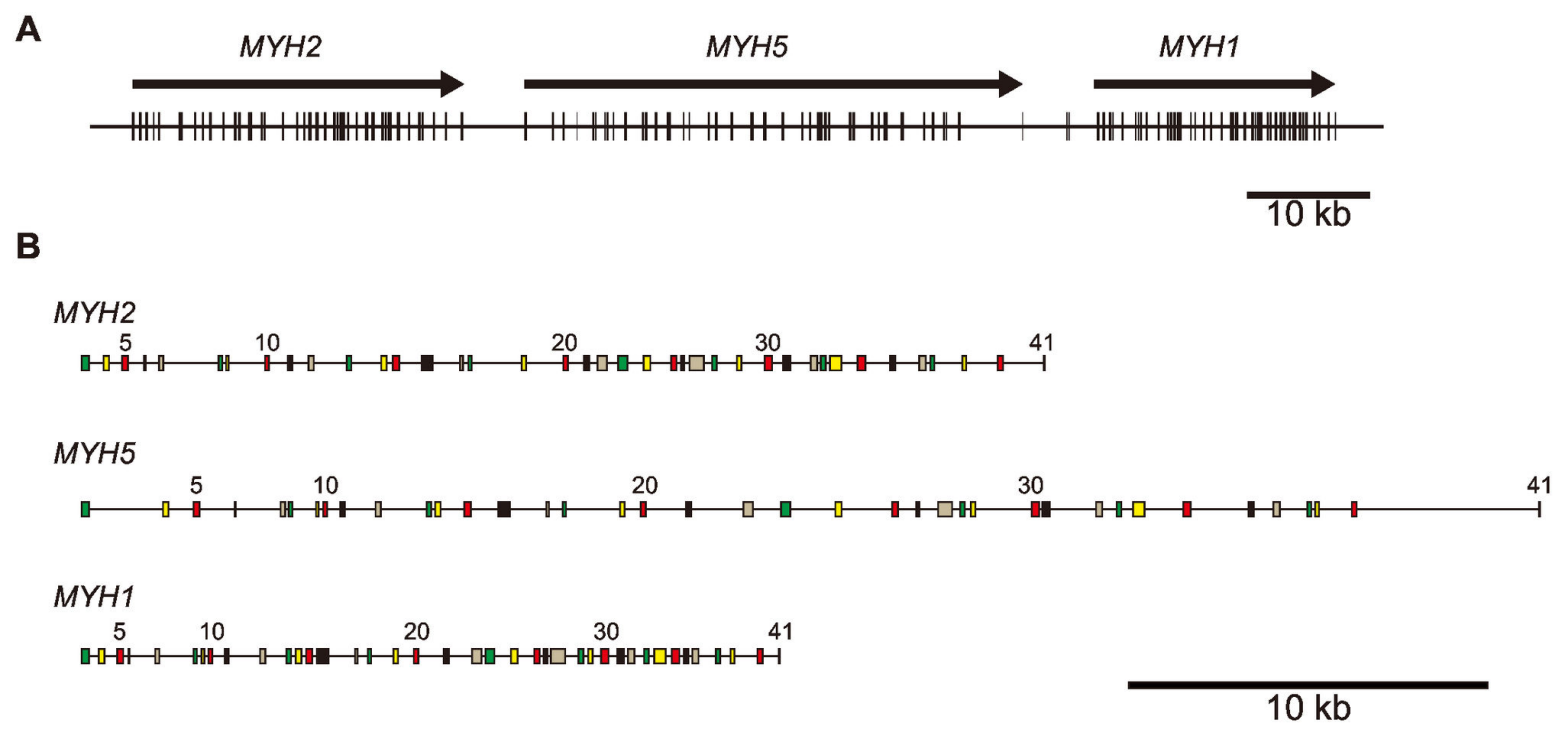
Physical map and gene organization of the Japanese lamprey fast skeletal myosin heavy chain genes (*MYH*s). (A) The position, orientation, size and gene organization of fast skeletal *MYH*s that compose the cluster. The vertical and horizontal lines indicate exons and introns, respectively. (B) The detailed gene organization of *MYH*s. Note that only the positions of coding exons 3-41 are presented. The boxes and lines indicate exons and introns, respectively.

 As shown in Figure S2, the sequences obtained from the sea lamprey genome database only fragmentarily covered the *MYH* cluster region. To analyze the full length of this region, we sequenced a 106-kb region containing all 3 fast skeletal *MYH*s of Japanese lamprey. Fourteen fragments in the *MYH* cluster region were amplified by the long PCR method (PCR primer sequences, their target regions and product sizes are listed in Table S1), and DNA sequences of respective fragments totally covering the full length of the *MYH* cluster region were determined by shotgun sequencing (AB720829). The resulting physical map and gene organization of *MYH2*-*MYH5*-*MYH1* are shown in Figure 1.

 Detailed analysis revealed that Japanese lamprey *MYH*s *1*, 2 and 5, each consisting of 39 coding exons, were 20 kb, 27 kb and 41 kb in length (start to stop codon), respectively (Figure 1 and Table S3). As shown in Table S3, the exon structures of fast skeletal *MYH*s of Japanese lamprey, humans and teleosts are highly conserved. The pattern of split codons (intron phase) is identical among all fast skeletal *MYH*s. Moreover, the respective exon sizes are identical except in 4 regions (exons 3, 7, 16-17 and 40-41) (Table S3). As reported previously, exons 3 and 40/41 encode the N- and C-termini, respectively [36]. The C-terminal exon 40 in human *MYH*s *1*, *2*, 4 and 8 corresponds to exons 40-41 in human *MYH*s 3 and 13 and teleost *MYH*s (Table S3) [36]. All Japanese lamprey *MYH*s possess exons 40-41, as is the case in human *MYH*s 3 and 13 and teleost *MYH*s (Table S3). The varied sizes of exons 7 and 16-17 encode variable lengths of loops 1 and 2, respectively [1,36].

 Although Japanese lamprey and sea lamprey are closely related, their genomic sequences are not identical. Therefore, we compared coding sequences of each *MYH* from Japanese lamprey and sea lamprey. The alignment used to calculate the sequence identities is provided in FASTA format (Dataset S1). Due to the lack of sea lamprey genome sequences, exons 12-20, 23-26 and 35-37 of *MYH1*; exons 26 and 35 of *MYH2*; and exons 7-9, 17, 18, 20, 27 and 32 of *MYH5* were excluded from analysis. The coding sequence identities of Japanese lamprey and sea lamprey *MYHs 1*, 2 and 5 are 97.1, 97.3 and 96.2%, respectively.

 During manuscript preparation, Mehta et al. [28] published and released the whole genome sequence of the Japanese lamprey. We found that scaffold00065 contained the fast skeletal *MYH* cluster (Figure S1). However, due to sequence gap regions, exons 2-15 of *MYH1* and exons 6, 17 and 21-23 of *MYH5* were not found in scaffold00065. We also found that *MAP2K4* and *GAS7* are located next to the 5′-site and 3′-site of the fast skeletal *MYH* cluster, respectively (Figure S1). *MAP2K4* is also located to the 5′-site of the fast skeletal *MYH* cluster of tetrapods and the 5′-site of non-clustered *MYH*s of teleosts [18] (Figure S1).

### Expression Analysis of Japanese Lamprey *MYH*s

 Although RT-PCR was carried out to detect the expression of Japanese lamprey *MYH5*, no transcript was observed in adult muscle or whole embryos at developmental stages 21, 25 and 27 (Figure S3). In contrast, the primers used for *MYH1*, *MYH2* and cytoplasmic actin *LjCA1* could detect the transcripts in all samples tested (Figure S3). 

### Phylogenetic Analysis

**Figure 2:**
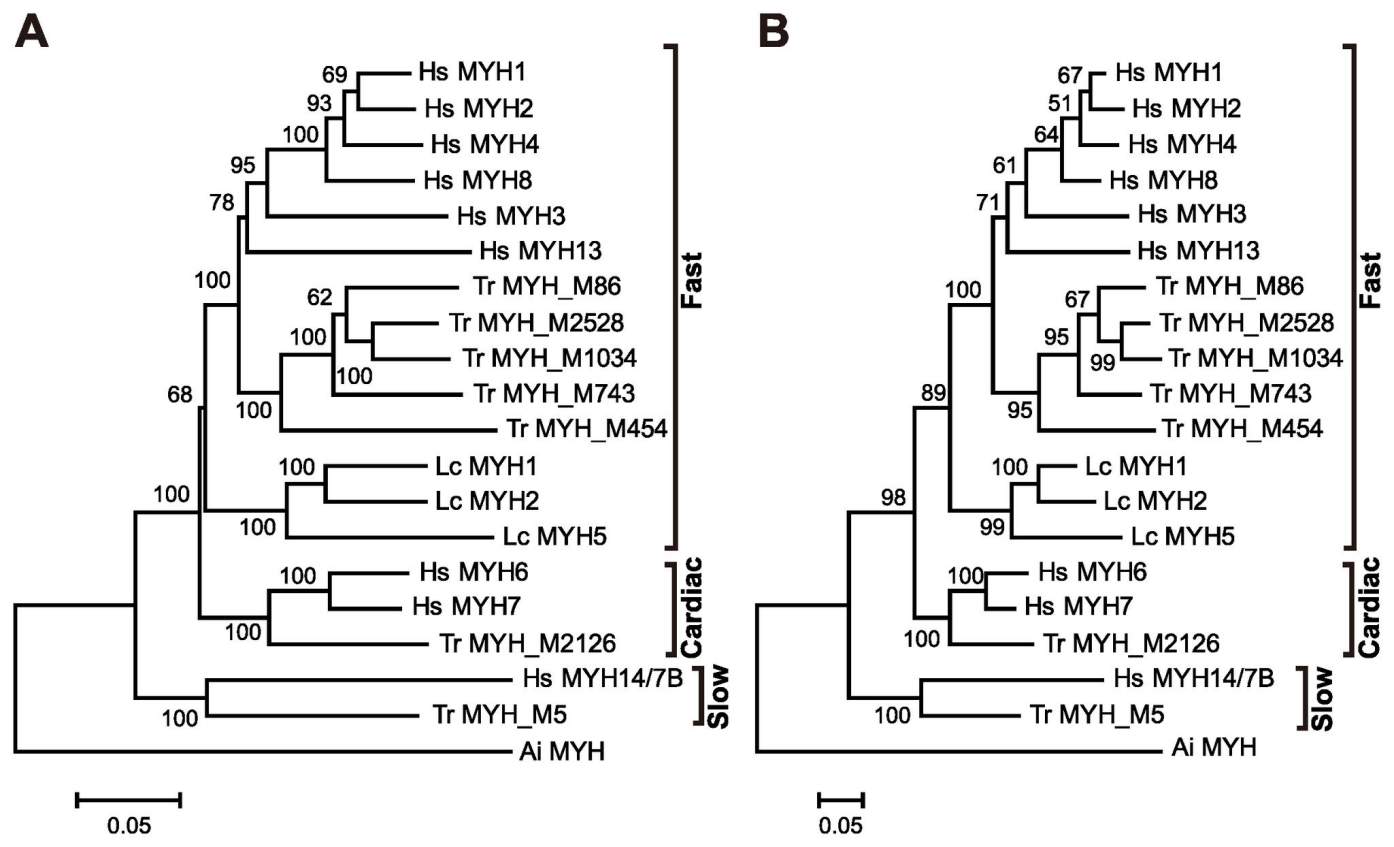
Phylogenetic analysis of myosin heavy chains (MYHs) from Japanese lamprey, torafugu and humans. The neighbor-joining (A) and maximum-likelihood (B) trees based on the full-length amino acid sequences of fast skeletal MYHs from Japanese lamprey, torafugu and humans. Cardiac and slow skeletal MYHs of torafugu and humans and sarcomeric MYH of bay scallops were used as outgroups. Lc MYHs from the Japanese lamprey *Lethenteron camtschaticum* were compared with Hs MYH1 (adult fast IId/x), Hs MYH2 (adult fast IIa), Hs MYH8 (perinatal fast), Hs MYH4 (adult fast IIb), Hs MYH3 (embryonic fast), Hs MYH13 (extraocular fast), Hs MYH6 (alpha cardiac), Hs MYH7 (beta cardiac) and Hs MYH14/7B (slow A) from human, *Homo sapiens*, and Ai MYH (striated muscle) from the bay scallop, *Argopecten irradians*. MYH sequences of the torafugu, *Takifugu rubripes* (Tr), were cited from our previous data [16-19].

 The full-length amino acid sequences deduced from Japanese lamprey and human *MYH*s were subjected to phylogenetic analysis. We used amino acid sequences of Japanese lamprey MYHs deduced from the full length of the *MYH* cluster region (AB720829). Because the full sequences of fast A (Tr MYH_M86)-, B (Tr MYH_ M2528 and M1034)-, C (Tr MYH_M743)- and D (Tr MYH_M454)-type *MYH*s were only available from torafugu [18], these were cited as references (Figure 2). In addition, the cardiac and slow skeletal MYHs of humans and torafugu and the sarcomeric MYH of bay scallop were used as outgroups.

 The alignment used to construct the phylogenetic tree is provided in FASTA format (Dataset S2). The topology obtained from the neighbor-joining tree is identical to that obtained from the maximum-likelihood tree, implying that the trees obtained are highly reliable (Figure 2). Phylogenetic analysis indicated that the 3 Japanese lamprey MYHs, including the newly identified Lc MYH5, are classified as a fast skeletal type but not as cardiac or slow skeletal types (Figure 2). Fast skeletal MYHs of humans, torafugu and Japanese lamprey formed 2 major clades, gnathostomes and cyclostomes, in the phylogenetic tree obtained in the present study (Figure 2). Therefore, there is no strict orthology between any Japanese lamprey *MYH* and any gnathostome *MYH*.

### Expression of Transgenes Containing the 5′-Flanking Sequences of Japanese Lamprey *MYH*s in Zebrafish

**Figure 3:**
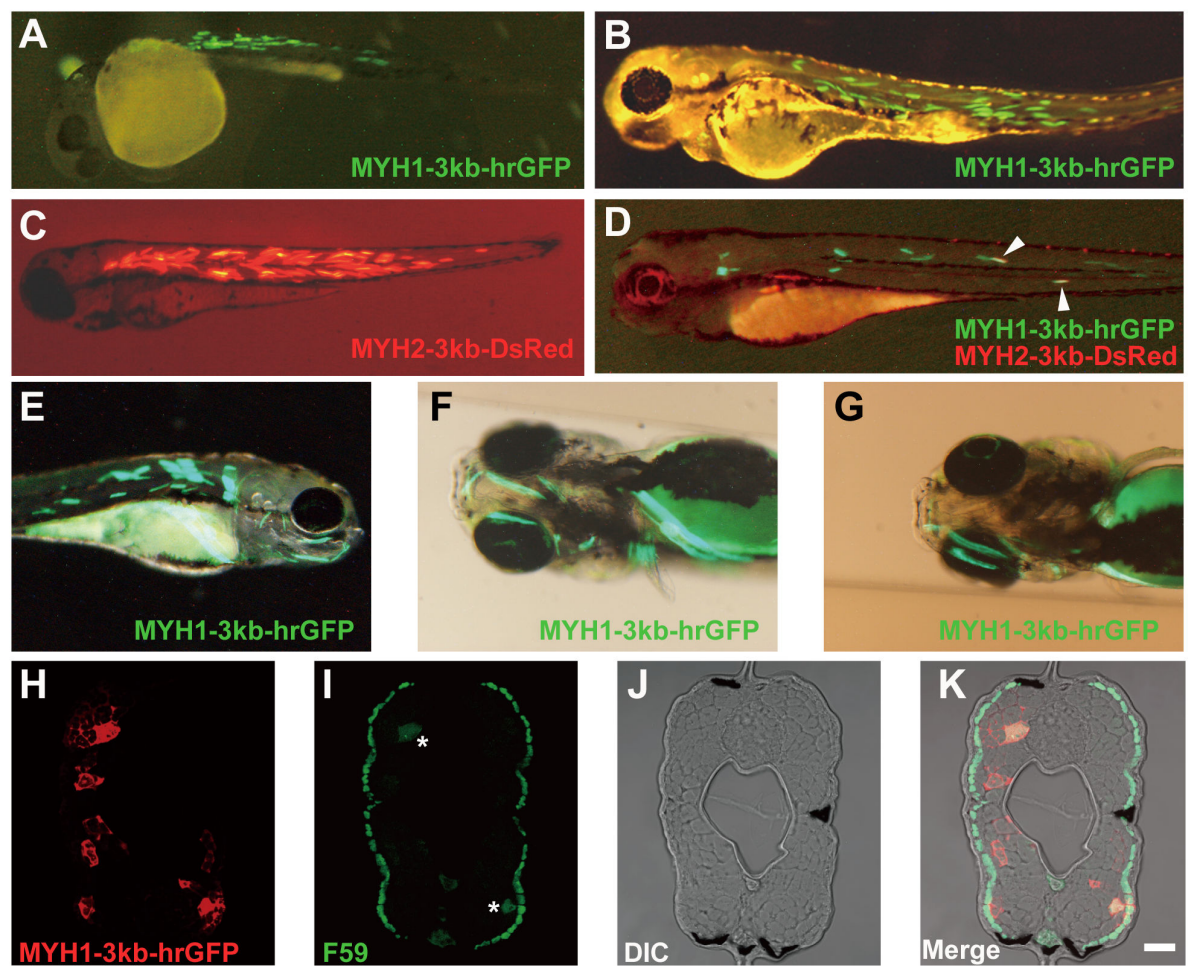
Transient expression of the Japanese lamprey MYH1-3kb-hrGFP and MYH2-3kb-DsRed transgenes in zebrafish embryos. A, B: Lateral view showing the expression of the MYH1-3kb-hrGFP in the trunk myotome of zebrafish embryos at 1 (A) and 3 (B) days post-fertilization (dpf). C: Lateral view showing the expression of the MYH2-3kb-DsRed in the trunk myotome of an embryo at 3 dpf. D: Lateral view showing the expression of the MYH1-3kb-hrGFP and MYH2-3kb-DsRed in the trunk myotome of an embryo at 3 dpf. Two muscle fibers clearly co-expressed the 2 transgenes in the same cells (merged, arrowheads). E-G: Lateral (E) and right (F) and left (G) ventrolateral side views showing the expression of the MYH1-3kb-hrGFP in cranial muscles of an embryo at 5 dpf. H-K: Transverse sections at the middle trunk of an embryo at 5 dpf. Cells were labeled with an antibody against GFP (H) and F59 antibody raised against chicken slow skeletal MYH (I). Differential interference contrast (DIC) microscopy image (J) and merged image from panels H, I and J (K). Asterisks indicate crosstalk signals due to strong GFP expression in the red channel. Note that superficial slow skeletal muscle fibers were labeled with F59, and the MYH1-3kb-hrGFP transgene was found to be expressed in fast muscle fibers. Scale bar = 20 μm.

 The 5′-flanking sequences of the medaka muscle actin genes are known to drive the expression of the reporter gene encoding green fluorescent protein (GFP) in the striated muscles of Japanese lamprey embryos [37]. These results imply that the 5′-flanking sequences of Japanese lamprey muscle-specific *MYH*s also drive the reporter gene expression in teleosts. To examine this possibility, the 5′-flanking sequences of Japanese lamprey fast skeletal *MYH*s were fused to the gene encoding GFP or DsRed and introduced into fertilized zebrafish eggs by microinjection. We used the regions of approximately 5, 3 and 2 kb from the start codon for Japanese lamprey *MYH*s *1*, *2* and *5*. In the case of *MYH*s 1 and 2, all the sequences functioned as the promoter with comparable activity in zebrafish muscle fibers (Figures 3 and S4 and Table S4). Furthermore, no obvious ectopic expression was observed. Therefore, we selected the sequence containing 3 kb from the start codon for further analysis. In addition, the regions -5, -3 and -2 kb from *MYH5* were unable to function as the promoter (Table S4).

 The -3 kb 5′-flanking sequences of Japanese lamprey *MYH1* (MYH1-3kb-hrGFP) and *MYH2* (MYH2-3kb-DsRed) were able to drive the transient expression of GFP and DsRed, respectively, both in the epaxial and hypaxial trunk muscles at 1 and 3 days post-fertilization (dpf) (Figure 3A-C). Furthermore, the MYH1-3kb-hrGFP and MYH2-3kb-DsRed transgenes were co-expressed in at least 2 muscle fibers in the same cells (merged in Figure 3D, arrowheads). The MYH1-3kb-hrGFP transgene was expressed in trunk muscles as well as in cranial muscles (Figure 3E-G). Immunohistological observations were then conducted to localize the muscle fiber types that express the reporter gene. An embryo expressing the MYH1-3kb-hrGFP transgene was stained with an antibody against GFP and F59 antibody raised against chicken slow skeletal MYH [38]. As shown in Figure 3K, superficial slow muscle fibers were labeled by F59, and the MYH1-3kb-hrGFP transgene was found to be expressed in fast muscle fibers.

### Expression of Transgenes Containing the 5′-Flanking Sequences of Japanese Lamprey *MYH*s in Mouse Myoblasts

**Figure 4:**
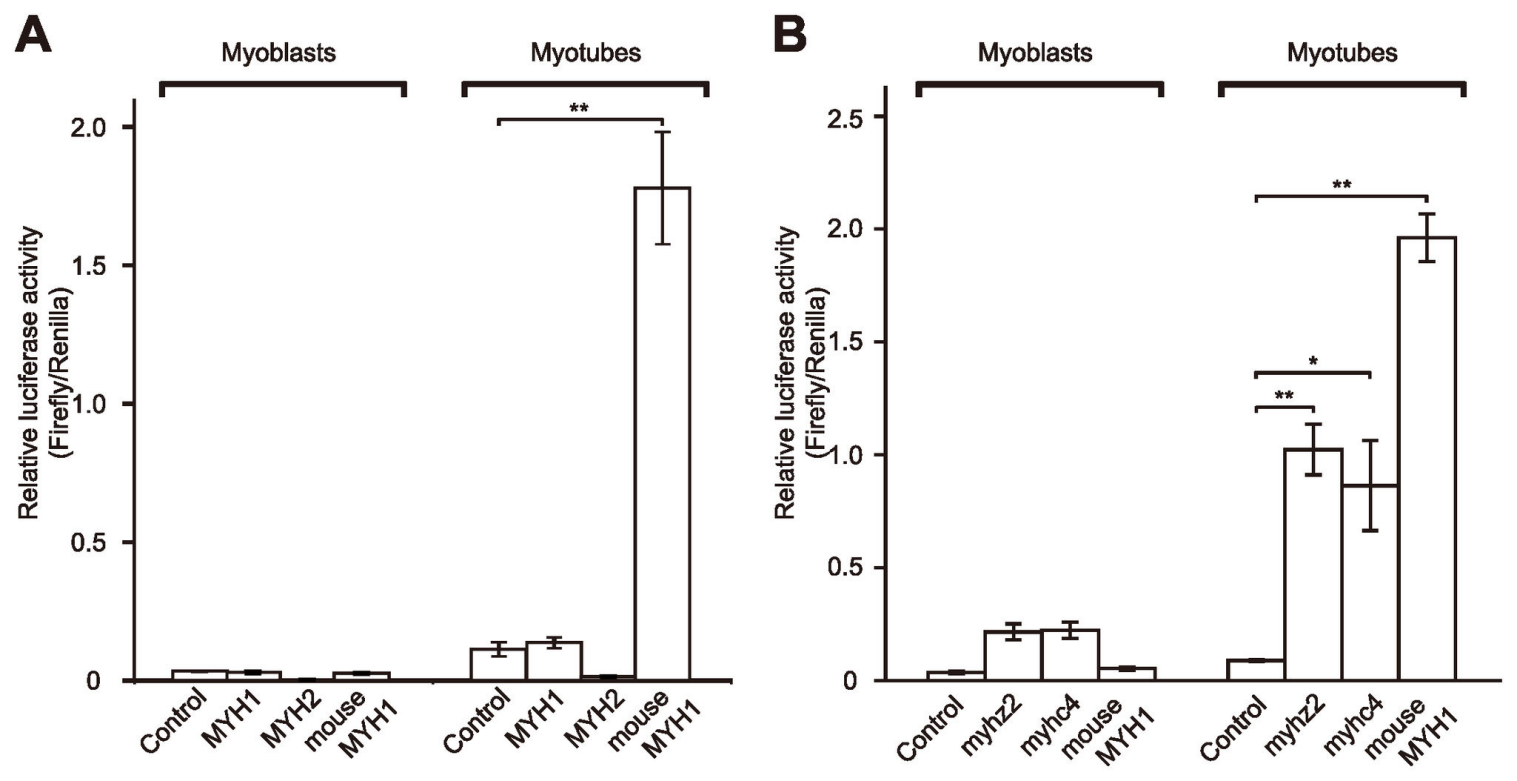
Activity of Japanese lamprey, zebrafish and mouse myosin heavy chain gene (MYH) promoter in C2C12 cells. A: C2C12 myoblasts were transfected with a promoter-less control pGL3 vector (control), -3 kb Japanese lamprey MYH1 (MYH1) or MYH2 (MYH2) and -2.8 kb mouse MYH1 (IId/x) (mouse MYH1) promoter luciferase constructs along with pRL-TK as a transfection control. B: C2C12 myoblasts were transfected with a promoter-less control pGL3 vector (control), -3 kb zebrafish *myhz2* (myhz2) or *myhc4* (myhc4) and -2.8 kb mouse MYH1 (IId/x) (mouse MYH1) promoter luciferase constructs along with pRL-TK as a transfection control. Cells were grown for 24 h in a growth medium, and myoblasts were harvested for luciferase assays. Other cells were cultured for an additional 4 days in differentiation medium, and myotubes were harvested for luciferase assays. The results are expressed as firefly luciferase activity per Renilla luciferase activity from each sample. The data represent the average of triplicate data, and all bars are the mean ± SD. Data were analyzed using Student's t test (*, P < 0.05; **, P < 0.01).

 To examine the promoter activity of Japanese lamprey *MYHs* in tetrapod muscles, the -3 kb 5′-flanking sequences of Japanese lamprey *MYH*s 1 and 2 were fused to the luciferase gene, introduced into mouse myogenic C2C12 cells and examined for luciferase activity. As previously reported [32], the activity of the mouse -2.8 kb *MYH1* promoter connected to the luciferase gene was significantly higher (70-fold) in differentiated myotubes than in proliferating myoblasts (Figure 4A). However, the luciferase activity of the constructs containing Japanese lamprey *MYH* 1 and 2 promoters were almost the same as or less than that of a promoter-less control pGL3 vector in myotubes (Figure 4A). In contrast, the zebrafish -3 kb *myhz2* [10] and *myhc4* [11] promoters had significant activity, approximately one-half that of the mouse -2.8 kb *MYH1*, in myotubes (Figure 4B).

### Conserved Non-coding Elements (CNEs) in the 5′-Flanking Sequences of Vertebrate *MYH*s

 The 5′-flanking sequences of cyclostome Japanese lamprey *MYH*s 1 and 2 fused to the reporter gene drove specific reporter gene expression in the fast skeletal muscle of zebrafish embryo (Figure 3), suggesting that the regulatory mechanisms involved in the transcriptional expression of fast skeletal *MYH*s are conserved between cyclostomes and teleosts. However, in contrast to the case of the proximal promoter of mouse *MYH1* and zebrafish *myhz2* and *myhc4*, the Japanese lamprey *MYH* promoters did not direct the reporter gene expression in differentiated mouse C2C12 myotubes (Figure 4). These results suggest that the muscle-specific regulatory mechanisms are partially conserved between teleosts and tetrapods but not between cyclostomes and tetrapods. To identify candidates for functionally conserved non-coding elements (CNEs) for muscle-specific expression between cyclostomes, teleosts and tetrapods, the 5′-flanking sequences of the *MYH*s of Japanese lamprey (the -3 kb *MYH*s 1 and 2 promoters used for the transgenic and the transfection assays), zebrafish (the -3 kb *myhz2* and *myhc4* promoters used for the transfection assay) and mouse (the -2.8 kb *MYH1* promoter used for the transfection assay) were aligned with Shuffle-LAGAN [33] and visualized with mVISTA [34] (Dataset S3).

 Shuffle-LAGAN alignment did not identify even coding exons between Japanese lamprey and mouse *MYH* sequences (Figure S5). When the promoters of Japanese lamprey *MYH2*, zebrafish *myhz2* and *myhc4* were aligned with that of Japanese lamprey *MYH1* as a baseline, we found 7, 11 and 11 CNEs, respectively (Figure S5A). Moreover, when the promoters of zebrafish *myhz2* and *myhc4* were aligned with that of mouse *MYH1* as a baseline, we found 4 and 7CNEs, respectively (Figure S5B). The identified CNEs were analyzed for conserved putative transcription factor binding sites (TFBSs) using the MATCH program based on the TRANSFAC database [35]. A cut-off to minimize false negative matches (minFN) was applied for searching putative TFBSs, as the minFN cut-off is useful for the detailed analysis of relatively short DNA fragments (see the on-line help information for the MATCH program) [35]. We found several conserved TFBSs in the CNEs, whereas the typical muscle-specific TFBSs, such as MyoD and myogenin, were not identified (Table S5). In contrast, conserved TFBSs for FOXD3 and Oct-1/POU2F1 were clearly identified (Table S5). We identified 10 FOXD3 and 5 Oct-1/POU2F1 binding sites in the Japanese lamprey/zebrafish CNEs (Table S5B, C). However, only 2 out of 10 FOXD3 and 2 out of 5 Oct-1/POU2F1 binding sites were identified with relatively high probability scores (core similarity >0.9, matrix similarity >0.85) (Tables S5B and C). By contrast, the FOXD3 binding site was not identified in the mouse/zebrafish CNEs (Table S5D, E). Although the probability score was not high, we identified 7 Oct-1/POU2F1 binding sites in the mouse/zebrafish CNEs (Table S5D, E).

## Discussion

 Like teleost and tetrapod *MYH*s, lamprey fast skeletal *MYH*s are tandemly arrayed (Figures 1, S1 and S2). Moreover, taking into consideration the synteny of lamprey *MYH*s with tetrapod and teleost counterparts, it was predicted that vertebrate *MYH*s evolved from a common ancestor of cyclostomes and gnathostomes. This hypothesis is strongly supported by the fact that the exon structure of fast skeletal *MYH*s is highly conserved between cyclostomes and gnathostomes (Table S3). Furthermore, all fast skeletal *MYH*s of Japanese lamprey and teleosts, as well as human *MYH*s 3 and 13, are encoded by 41 exons, implying that the common ancestor of vertebrate fast skeletal *MYH* was encoded by 41 exons. Human *MYH*s *1*, *2*, 4 and 8, which are encoded by 40 exons, are likely to have lost intron 40 after divergence from the common ancestor of human *MYH3* and *MYH*s *1*, *2*, *4* and *8* (Figure 2).

 The newly identified Japanese lamprey *MYH5* is not expressed either in whole embryo or adult muscle tissue (Figure S3). However, a phylogenetic analysis indicated that the 3 Japanese lamprey MYHs, including the newly identified MYH5, were classified as a fast skeletal type but not as cardiac or slow skeletal types (Figure 2). Moreover, in the present study, strict orthology was not found in *MYH*s between cyclostomes and gnathostomes (Figure 2). It is possible that the tandem duplication of fast skeletal *MYH* occurred in the lamprey lineage after splitting from the gnathostome lineage and that fast skeletal *MYH* clustering was not an early event in vertebrate evolution. Indeed, the phylogenetic tree indicates that there was a single fast skeletal *MYH* in the common ancestor of lampreys, teleost fish and mammals and that the *MYH* underwent independent duplications in the different lineages (Figure 2). Although other chordate lineages, such as the amphioxus *B. floridae* and the tunicate *C. savignyi*, do possess at least 1 *MYH* cluster (Figure S1), no conserved synteny was found between vertebrate and other chordate *MYH* clusters (Figure S1). In addition, McGuigan et al. have shown that tunicate *MYH*s are phylogenetically distinct from diverse *MYH*s in vertebrates [12]. However, *MYH* clusters obviously exist in amphioxus and tunicates, implying that the formation of *MYH* clusters was a common event and is not a novel feature established in the vertebrate lineage but rather is shared by the broader taxa of animals. The functional significance in the formation of *MYH* clusters for chordate evolution remains an open question.

 Kusakabe et al. [37] showed that the GFP reporter gene was expressed predominantly in the striated muscles of Japanese lamprey embryos when driven by the 5′-flanking sequence of the medaka muscle actin gene. Accordingly, the authors hypothesized that the regulatory mechanisms involved in the muscle-specific gene expression had evolved before the divergence between cyclostomes and gnathostomes [37]. Our finding that the 5′-flanking sequence of cyclostome Japanese lamprey *MYH*s 1 and 2 fused to the reporter gene drove the expression of the reporter gene in the fast skeletal muscle of gnathostome zebrafish (see Figure 3) clearly supports their hypothesis. The transcripts of Japanese lamprey *MYH*s 1 and 2 showed an almost identical spatiotemporal expression pattern in whole-mount *in situ* hybridization [23]. Similarly, we found no apparent differences in the expression patterns of the reporter genes driven by *MYH*s *1* and *2* (see Figure 3A-C). In fact, the MYH1-3kb-hrGFP and MYH2-3kb-DsRed transgenes were co-expressed in at least 2 muscle fibers in the same cells (see Figure 3D). These results suggest that the regulatory mechanisms involved in the transcriptional expression of fast skeletal *MYH*s are conserved between cyclostomes and teleosts. However, it should also be noted that the expression patterns of Japanese lamprey fast skeletal *MYH1* do not precisely correspond to the spatial location of muscle fiber types found in gnathostomes such as zebrafish. At embryonic stage 25, *MYH1* (*LjMyHC1*) is expressed in the myotomal cells except the medial part of the myotome adjacent to the notochord, which might represent the Japanese lamprey adaxial cells [39]. Later, at stage 28, *MYH1* is expressed in the adaxial cells but not in the deeper region of the somite [39]. It is well known that the adaxial cells are fated to give rise to slow skeletal muscle fiber in zebrafish [39]. Therefore, according to the endogenous expression pattern, Japanese lamprey fast skeletal *MYH1* has the potential to be expressed both in slow and fast skeletal muscle fibers. To address whether the regulatory mechanisms involved in the transcriptional expression of fast skeletal *MYH*s are exactly conserved between cyclostomes and teleosts, the establishment of stable transgenic lines is needed. It was also noted that Japanese lamprey *MYH1* promoter drove GFP expression in zebrafish cranial muscle derived from the head mesoderm, such as the intermandibularis anterior, intermandibularis posterior and interhyoideus (see Figure 3E-D) [7,40,41]. This pattern is in marked contrast to the fact that the expression of *MYH*s 1 and 2 is absent in head mesoderm derivatives [23,39,42]. It is possible that the -3 kb 5′-flanking sequence of Japanese lamprey *MYH1* does not carry regulatory elements sufficient to recapitulate the endogenous *MYH1* expression. GFP reporter analyses on lamprey embryos will provide conclusive results.

 The present study reveals that there is obvious synteny between lamprey and tetrapod *MYH* cluster regions (see Figures S1 and S2). Thus, the 5′-flanking sequences of lamprey *MYH*s 1 and 2 were expected to drive the reporter gene expression in tetrapod myogenic cells. However, in contrast to the case of the proximal promoter of mouse *MYH1*, the Japanese lamprey *MYH* promoters did not direct the reporter gene expression in differentiated mouse C2C12 myotubes (see Figure 4A). These results suggest that although the common ancestor of teleosts and tetrapods shared the same muscle-specific regulatory mechanisms, tetrapods have not retained these regulatory mechanisms.

 It was evident that the proximal promoters of Japanese lamprey fast skeletal *MYH*s drove the reporter gene expression in fast skeletal fibers of zebrafish embryos where endogenous *MYH*s were expressed. In zebrafish, at least 3 fast skeletal *MYH*s, *myhz1*, *myhz2* and *myhc4*, are dominantly expressed in fast fibers of embryos [9-11]; orthologs of torafugu [18], medaka [14] and common carp [7,8] are also expressed in embryos from the respective fish species. In addition, the proximal promoters of the above-listed *MYH*s from torafugu [43,44] and medaka [15] drive the reporter gene expression in the fast fibers of zebrafish embryos. These teleost *MYH*s are all classified as fast C type [18], suggesting that the proximal promoters of fast skeletal *MYH*s of lampreys and fast C-type *MYH*s of teleosts have a common ancestor and share common regulatory mechanisms, even though no synteny (see Figures S1 and S2) [18] or strict orthology (see Figure 2) was found between lamprey and teleost *MYH*s. Indeed, we found CNEs between promoters of Japanese lamprey *MYH*s 1 and 2 as well as between Japanese lamprey *MYH1* and zebrafish *myhz2* and *myhc4* (Figure S5A). As the zebrafish *myhz2* and *myhc4* are among the dominantly expressed *MYH*s in fast fibers of embryos [9-11], these CNEs have the potential to direct gene expression in zebrafish embryos. We identified 10 FOXD3 and 5 Oct-1/POU2F1 binding sites in the Japanese lamprey/zebrafish CNEs (Table S5B, C).

 In zebrafish, FOXD3 mediates the expression of *myf5*, one of the muscle regulatory factors, and affects myogenesis [45]. Flounder FOXD3 is also involved in myogenesis by regulating the expression of *myf5* and *MyoD* [46]. Conserved TFBSs for FOXD3 are remarkably identified in the Japanese lamprey/zebrafish CNEs (Table S5B, C), implying that fast skeletal *MYH*s of lamprey and zebrafish are also regulated by FOXD3. Moreover, the muscle-specific expression of fast skeletal *MYH*s in mice is at least partly controlled by the transcription factors MEF2 and Oct-1/POU2F1 [47,48]. However, only 2 out of 10 FOXD3 and 2 out of 5 Oct-1/POU2F1 binding sites in the Japanese lamprey/zebrafish CNEs were identified with high probability scores (core similarity >0.9, matrix similarity >0.85) (Tables S5B and C). Therefore, transgenic analysis using the deletion constructs of these CNEs is needed to evaluate the significance of these CNEs for gene expression in zebrafish.

 To determine whether the regulatory mechanisms involved in the transcriptional expression of fast skeletal *MYH*s are conserved between teleosts and tetrapods, the promoter activities of zebrafish *myhz2* and *myhc4* were examined in C2C12 myogenic cells (Figure 4B). In contrast to Japanese lamprey promoters, the zebrafish promoters exhibited significant activity in differentiated mouse C2C12 myotubes (Figure 4B). In fact, Shuffle-LAGAN alignment did not identify even coding exons between Japanese lamprey and mouse *MYH* sequences (Figure S5). These results suggest that the muscle-specific regulatory mechanisms are partially conserved between teleosts and tetrapods but not between lampreys and tetrapods.

 We found 11 CNEs conserved between promoters of mouse and zebrafish *MYH*s (Figure S5B). As the zebrafish promoters exhibited significant activity in differentiated mouse C2C12 myotubes (Figure 4B), these CNEs have the potential to direct the reporter gene expression in tetrapods. In contrast to the mouse/zebrafish CNEs, the FOXD3 binding site was not identified, but 7 Oct-1/POU2F1 binding sites were identified (Table S5D, E). This finding implies that Oct-1/POU2F1 binding sites, rather than FOXD3 binding sites, are important for the transcriptional expression of fast skeletal *MYH*s in tetrapods. However, the probability scores of Oct-1/POU2F1 binding sites in the mouse/zebrafish CNEs were not significant (Table S5D, E). Again, transfection analysis using the deletion constructs of these CNEs is needed to evaluate the significance of these CNEs for the gene expression in mouse C2C12 cells. However, our conclusion that tetrapods do not retain the same muscle-specific regulatory mechanisms as in teleosts is based on the finding that Japanese lamprey *MYH* promoters were not activated in C2C12 myogenic cells. Naturally, gene sets transcribed in the myogenic cell lines are different from those transcribed in myotomes of developing mammalian embryos. LacZ reporter analyses on mouse embryos will provide conclusive results.

## Supporting Information

Table S1 **PCR primer sequences, their target regions and product sizes.**
(DOCX)

Table S2 **Nucleotide sequence of primers used in the present study.**
(DOCX)

Table S3 **Intron phases and the coding exon sizes of vertebrate myosin heavy chain genes (*MYH*s).**
(DOCX)

Table S4 **Quantification of GFP or DsRed expressing zebrafish embryos.**
(DOCX)

Table S5 **Conserved non-coding elements (CNEs) identified by Shuffle-LAGAN.**
(DOCX)

Figure S1 **Physical maps of Japanese and sea lampreys, human and torafugu syntenic regions containing fast skeletal myosin heavy chain genes (*MYH*s).** *Branchiostoma floridae* and *Ciona savignyi* genomic regions containing sarcomeric *MYH*s are also presented. Gray and open rectangles indicate *MYH*s and non-syntenic genes, respectively. Syntenic genes *MAP2K4, TMEM220, ADPRM, SCO1* and *GAS7* are color-coded. Arrows indicate the directions of gene transcription. Five non-syntenic genes between human *MAP2K4* and *TMEM220* are not shown. Note that as the presence of other genes between *MYH1* and *GAS7* remains a possibility in the sea lamprey genome, the intergenic region is shown by a dashed line. Asterisks (*) indicate that there is no orthologous relationship between lamprey and human *MYH1*/*MYH2*.
(EPS)

Figure S2 **Schematic representation of the location of the sea lamprey myosin heavy chain genes (*MYH*s) and growth arrest-specific protein 7 gene (*GAS7*) on the genome.** The relative positions of contigs and a scaffold are shown. Contigs 2034, 15297, 6974, 38608 and 39193 and scaffold GL477325 were extracted from the sea lamprey genome databases Petromyzon_marinus-3.0 and -7.0, respectively. Arrows indicate the directions of transcription of the genes. A region with a long sequence gap in scaffold GL47325 is shown by a dashed line, and contigs 6974/38608 and 39193 are expected to be located on this gap region. At present (November 2013), the Petromyzon_marinus-3.0 database is obtainable from the Genome Institute at Washington University (http://genome.wustl.edu/pub/organism/Other_Vertebrates/Petromyzon_marinus/).
(EPS)

Figure S3 **Expression analysis of Japanese lamprey myosin heavy chain genes (*MYH*s).** The target regions for amplification of each *MYH* are shown in brackets. The gene encoding cytoplasmic actin LjCA1 was used as the positive control.
(TIF)

Figure S4 **Transient expression of the Japanese lamprey MYH1-2kb-hrGFP, MYH1-5kb-hrGFP, MYH2-2kb-DsRed and MYH2-5kb-DsRed transgenes in zebrafish embryos.** Lateral view showing the expression of the MYH1-2kb-hrGFP (A), MYH2-2kb-DsRed (B), MYH1-5kb-hrGFP (C) and MYH2-5kb-DsRed (D) in the trunk myotome of zebrafish embryos at 3 days post-fertilization. Arrowheads indicate autofluorescence due to pigment cells on the embryo surface.
(TIF)

Figure S5 **Shuffle-LAGAN alignment of the 5' flanking sequences of myosin heavy chain genes (*MYH*s).** The 5' flanking sequences of Japanese lamprey *MYH1* and *MYH2*, zebrafish *myhz2* and *myhc4* and mouse *MYH1* are aligned with Shuffle-LAGAN and visualized with mVISTA. The 5' flanking sequences of Japanese lamprey *MYH1* (A) and mouse *MYH1* (B) are used as baselines. Coding (blue) and non-coding (light blue) exons are annotated. Pink peaks represent non-coding conservation above 70% over at least 20 bp.
(TIF)

Dataset S1 **Coding sequences of Japanese lamprey and sea lamprey myosin heavy chain genes (*MYH*s) *1*, *2* and *5*.**
(TXT)

Dataset S2 **Amino acid sequence alignment of myosin heavy chains (MYHs) from Japanese lamprey, torafugu and humans.**
(TXT)

Dataset S3 **5′-flanking sequences of the myosin heavy chain genes (*MYH*s) of Japanese lamprey, zebrafish and mouse.**
(TXT)
