# Supplementary material for: Lampreys Have a Single Gene Cluster for the Fast Skeletal Myosin Heavy Chain Gene Family
Source: PLoS One. 2013 Dec 20;8(12):e85500. doi: 10.1371/journal.pone.0085500 (PMC3869912; doi:10.1371/journal.pone.0085500)
Supplement: Table S1 — PCR primer sequences, their target regions and product sizes. (DOCX) [file pone.0085500.s001.docx]

| Table S1. PCR primer sequences, their target regions and product sizes | | | |
| --- | --- | --- | --- |
| Target region | Primer name | Sequence | Product size (kb) |
| MYH2 5' flanking region - exon 3 | MYH2_5'UTR_Forward | TTCAAGTCCCACCAGCTGCCAAGGCGCAAATAC | 8.4 |
|  | MYH1/2_ex3_Reverse | GACGGTGACTTTGCCACCCTCGCTGCTCTTGATG |  |
| MYH2 exons 3-17 | MYH2_ex3_Forward | GCCCAGACTCGCCCCTTCGACATGAAG | 10.6 |
|  | MYH2_ex17_Reverse | GTGCAGGGCAGACACTGTCTGGAAGGAGGATCC |  |
| MYH2 exons 17-25 | MYH2_ex17_Forward | TTGTGTAGACACCGGCAGCAAGGGAGG | 6.0 |
|  | MYH2_ex25_Reverse | CTCTTGGTTCTTTCGACGTCCATGCGG |  |
| MYH2 exons 25-33 | MYH2_ex25_Forward | CGAGGGCTCGTTGGAGCAAGAGAAGAAG | 4.3 |
|  | MYH2_ex33_Reverse | CTCTTGTTCAAGCTGCTTACGGCTCTTCTCC |  |
| MYH2 exons 33-41 | MYH2_ex33_Forward | TTGGCGAGAGCGGAAAGGCCCTGCACGAGG | 6.3 |
|  | MYH2_ex41_Reverse | TGTGTAGATCCTAAGCAGCAGTAAAGTCAATCGC |  |
| MYH2 exon 41 - MYH3 exon 3 | MYH2_ex41_Forward | GCCGAGTAATGCCAAAGGTATCGAACTACCGTAGAG | 5.4 |
|  | MYH3_ex3_Reverse | CAACGGTGACTTTGCCACCGTCACTGCTCTTGACC |  |
| MYH3 exons 3-11 | MYH3_ex3_Forward | GATTGGAAGCTCAGCACCGTCCCTTTGACAC | 7.2 |
|  | MYH3_ex11_Reverse | TGCCATGAGCTCTTCTTTGTCATCAATAGATGCC |  |
| MYH3 exon 10 - intron 21 | MYH3_ex10_Forward | TCTGCTGGAGAAATCCCGTGTCACCTTCCAGC | 11.1 |
|  | MYH3_in21_Reverse | GGCCATGATTTGAACCCATGTCACCGTATTCATC |  |
| MYH3 exon 19 - intron 26 | MYH1/3_ex19_Forward | GATCTGCAGGAAGGGCTTCCCCAACAGG | 8.3 |
|  | MYH3_in26_Reverse | TACTTTTCCCCCCAGGGCACCCCTCGGTTG |  |
| MYH3 exon 26 - intron 40 | MYH3_ex26_Forward | ATGCAAGATGAAATGGCTCTCTCAGCTCAGCTAC | 12.9 |
|  | MYH3_in40_Reverse | GGGAATAGGGGGAAGTCTCTGTCTGAACACGTGG |  |
| MYH3 exon 40 - MYH1 exon 3 | MYH3_ex40_Forward | TGAGGGCCAAGTCTCGTGATGTGGCCAATCAAA | 11.4 |
|  | MYH1/2_ex3_Reverse | GACGGTGACTTTGCCACCCTCGCTGCTCTTGATG |  |
| MYH1 exons 3-10 | MYH1_ex3_Forward | CGCCTAGAAACCAGGCAAGATGGGAGACC | 3.6 |
|  | MYH1_ex10_Reverse | GGAAGAAGATGTGGTAGTTTCTCTCTGTTGG |  |
| MYH1 exons 10-21 | MYH1_ex10_Forward | TCTGCTGGAGAAATCCCGTGTCACCTTCCAGC | 6.7 |
|  | MYH1_ex21_Reverse | AGTCGGTCGTCCCGCATCTCCTCCAGGG |  |
| MYH1 exons 19-41 | MYH1/3_ex19_Forward | GATCTGCAGGAAGGGCTTCCCCAACAGG | 10.7 |
|  | MYH1_ex41_Reverse | CTTGAGTTGCATTGCTTGTGTGCAAAGGG |  |
