# Supplementary material for: Lampreys Have a Single Gene Cluster for the Fast Skeletal Myosin Heavy Chain Gene Family
Source: PLoS One. 2013 Dec 20;8(12):e85500. doi: 10.1371/journal.pone.0085500 (PMC3869912; doi:10.1371/journal.pone.0085500)
Supplement: Table S2 — Nucleotide sequence of primers used in the present study. (DOCX) [file pone.0085500.s002.docx]

| Table S2. Nucleotide sequence of primers used in the present study | |
| --- | --- |
|  | |
| Table S2A. Nucleotide sequence of primers used for RT-PCR | |
| Primer name | Sequence |
| MYH1_exon29_Forward | GAAAGCAGTCCTACACCCAGCAAG |
| MYH2_exon29_Forward | GGAAGCTGTCCTACACACAGCAGG |
| MYH5_exon29_Forward | TAAAACAGTGCAACAGCCAACAAA |
| MYH1_exon31_Forward | GTCTGCAGGGTGAAGTGGAG |
| MYH2_exon31_Forward | GTCTGCTGGGTGAAGTGGAG |
| MYH5_exon31_Forward | GTCTGCAAGCGGAGATCGAA |
| MYH1_exon33_Reverse | TGTTCTTCTCAAGCTCGTGCAGGC |
| MYH2_exon33_Reverse | GGCTCTTCTCCACCTCGTGCAGGG |
| MYH5_exon33_Reverse | TGTACTTGTCCAGCTCGTGTACAT |
| LjCA1_Forward | ACTCTGGTGACGGCGTGTCG |
| LjCA1_Reverse | GTTGAAGGTGGTCTCGTGAATG |
|  | |
| Table S2B. Nucleotide sequence of primers for amplification of the 5'-flanking region of *MYH*s | |
| Primer name | Sequence |
| MYH1_5k_SacII_Forward | ATGCGCCCGCGGCCATTGTGCCTCTGATGTCGTGC |
| MYH1_SacII_Reverse | ATGCGCCCGCGGCTTGCCTGGTTTCTAGGCGTG |
| MYH2_5k_BamHI_Forward | CGCGGATCCCACAGCTGTTTTATTGCCAGAAAG |
| MYH2_BamHI_Reverse | CGCGGATCCCTTGCCTGGTCTTTAGGTGT |
| MYH5_5k_BamHI_Forward | CGCGGATCCAAGGTATCGAACTACCGTAGAG |
| MYH5_BamHI_Reverse | CGCGGATCCGTTTGGCTGGCGATCCCTCAC |
|  | |
| Table S2C. Nucleotide sequence of primers to remove unnecessary regions for reporter gene expression | |
| Primer name | Sequence |
| MYH1_2k_Forward | GCAGCAAACTACTGCATAACCATCC |
| MYH1_3k_Forward | CCGCAGGCTACGAATTAAAGTTGTT |
| MYH1_5k_SacII_Forward | ATGCGCCCGCGGCCATTGTGCCTCTGATGTCGTGC |
| MYH2_2k_Forward | CCTGTTTCCAAAACGTCCAAAAAGT |
| MYH2_3k_Forward | GACGGGCTAAGGATTGAAACGTAAC |
| MYH2_5k_BamHI_Forward | CGCGGATCCCACAGCTGTTTTATTGCCAGAAAG |
| MYH5_2k_Forward | CTTTCAAAGGCAGCAGACAACCTAC |
| MYH5_3k_Forward | ATCAGCTCAGACCGAGGAGAAATTA |
| MYH5_5k_BamHI_Forward | CGCGGATCCAAGGTATCGAACTACCGTAGAG |
| f1_origin_Reverse | CCTGATAGACGGTTTTTCGCCCTTT |
|  | |
| Table S2D. Nucleotide sequence of primers for amplification of the 5'-flanking region of *MYH*s | |
| Primer name | Sequence |
| pGL3_infusion_Forward | AGCTTGGCATTCCGGTACTG |
| pGL3_infusion_Reverse | CTAGCACGCGTAAGAGCTCG |
| MYH2_3k_infusion_pGL3_Forward | TCTTACGCGTGCTAGCCTGCATATAAGGCGTGCAAGTG |
| MYH2_infusion_pGL3_Reverse | CCGGAATGCCAAGCTTCTTGCCTGGTCTTTAGGTGT |
| MYH1_3k_infusion_pGL3_Forward | TCTTACGCGTGCTAGCAAACTACGTGGCCAGGAATCG |
| MYH1_infusion_pGL3_Reverse | CCGGAATGCCAAGCTTCTTGCCTGGTTTCTAGGCGTG |
| Myhz2_3k_infusion_pGL3_Forward | TCTTACGCGTGCTAGCATAAGCTTGCCATCAAGATCAGG |
| Myhz2_infusion_pGL3_Reverse | CCGGAATGCCAAGCTTGGTGGCGGCTTACTAAAGAAAG |
| Myhc4_3k_infusion_pGL3_Forward | TCTTACGCGTGCTAGCTAGGTTGCTGCAATCAAGTTGTG |
| Myhc4_infusion_pGL3_Reverse | CCGGAATGCCAAGCTTGGTGGCGGCTTGGTGGAAATC |
| MYHIIdx_2.8k_infusion_pGL3_Forward | TCTTACGCGTGCTAGCTGTGGGTCACACAGTCCCTTG |
| MYHIIdx_infusion_pGL3_Reverse | CCGGAATGCCAAGCTTGGCTGCGGGCTATTGGTTGC |
