# Supplementary material for: Lampreys Have a Single Gene Cluster for the Fast Skeletal Myosin Heavy Chain Gene Family
Source: PLoS One. 2013 Dec 20;8(12):e85500. doi: 10.1371/journal.pone.0085500 (PMC3869912; doi:10.1371/journal.pone.0085500)
Supplement: Table S3 — Intron phases and the coding exon sizes of vertebrate myosin heavy chain genes (MYHs). (DOCX) [file pone.0085500.s003.docx]

| Table S3. Intron phases and the coding exon sizes of vertebrate myosin heavy chain genes *(MYH*s*)* | | | | | | | | | | | | |
| --- | --- | --- | --- | --- | --- | --- | --- | --- | --- | --- | --- | --- |
| Coding exon | Intron  phase | Exon size (bp) | | | | | | | | | | |
|  |  | Lamprey | | | Carp  MYH | Torafugu  MYH | Human | | | | | |
|  |  | MYH1 | MYH2 | MYH5 |  |  | MYH1 | MYH2 | MYH3 | MYH4 | MYH8 | MYH13 |
| 3* | 0 | 195 | 195 | 195 | 201 | 204 | 204 | 183 | 204 | 204 | 210 | 204 |
| 4 | 0 | 144 | 144 | 144 | 144 | 144 | 144 | 144 | 144 | 144 | 144 | 144 |
| 5 | 1 | 157 | 157 | 157 | 157 | 157 | 157 | 157 | 157 | 157 | 157 | 157 |
| 6 | 2 | 28 | 28 | 28 | 28 | 28 | 28 | 28 | 28 | 28 | 28 | 28 |
| 7* | 0 | 112 | 109 | 115 | 112 | 115 | 115 | 115 | 109 | 115 | 109 | 112 |
| 8 | 0 | 93 | 93 | 93 | 93 | 93 | 93 | 93 | 93 | 93 | 93 | 93 |
| 9 | 1 | 64 | 64 | 64 | 64 | 64 | 64 | 64 | 64 | 64 | 64 | 64 |
| 10 | 1 | 99 | 99 | 99 | 99 | 99 | 99 | 99 | 99 | 99 | 99 | 99 |
| 11 | 0 | 104 | 104 | 104 | 104 | 104 | 104 | 104 | 104 | 104 | 104 | 104 |
| 12 | 1 | 139 | 139 | 139 | 139 | 139 | 139 | 139 | 139 | 139 | 139 | 139 |
| 13 | 0 | 119 | 119 | 119 | 119 | 119 | 119 | 119 | 119 | 119 | 119 | 119 |
| 14 | 0 | 150 | 150 | 150 | 150 | 150 | 150 | 150 | 150 | 150 | 150 | 150 |
| 15 | 0 | 171 | 171 | 171 | 171 | 171 | 171 | 171 | 171 | 171 | 171 | 171 |
| 16* | 1 | 310 | 310 | 310 | 301 | 301 | 310 | 310 | 307 | 310 | 310 | 310 |
| 17* | 0 | 65 | 68 | 68 | 68 | 77 | 71 | 68 | 71 | 71 | 68 | 74 |
| 18 | 1 | 88 | 88 | 88 | 88 | 88 | 88 | 88 | 88 | 88 | 88 | 88 |
| 19 | 2 | 118 | 118 | 118 | 118 | 118 | 118 | 118 | 118 | 118 | 118 | 118 |
| 20 | 0 | 124 | 124 | 124 | 124 | 124 | 124 | 124 | 124 | 124 | 124 | 124 |
| 21 | 2 | 137 | 137 | 137 | 137 | 137 | 137 | 137 | 137 | 137 | 137 | 137 |
| 22 | 0 | 256 | 256 | 256 | 256 | 256 | 256 | 256 | 256 | 256 | 256 | 256 |
| 23 | 0 | 243 | 243 | 243 | 243 | 243 | 243 | 243 | 243 | 243 | 243 | 243 |
| 24 | 0 | 177 | 177 | 177 | 177 | 177 | 177 | 177 | 177 | 177 | 177 | 177 |
| 25 | 2 | 146 | 146 | 146 | 146 | 146 | 146 | 146 | 146 | 146 | 146 | 146 |
| 26 | 0 | 91 | 91 | 91 | 91 | 91 | 91 | 91 | 91 | 91 | 91 | 91 |
| 27 | 0 | 390 | 390 | 390 | 390 | 390 | 390 | 390 | 390 | 390 | 390 | 390 |
| 28 | 1 | 127 | 127 | 127 | 127 | 127 | 127 | 127 | 127 | 127 | 127 | 127 |
| 29 | 0 | 119 | 119 | 119 | 119 | 119 | 119 | 119 | 119 | 119 | 119 | 119 |
| 30 | 2 | 197 | 197 | 197 | 197 | 197 | 197 | 197 | 197 | 197 | 197 | 197 |
| 31 | 0 | 184 | 184 | 184 | 184 | 184 | 184 | 184 | 184 | 184 | 184 | 184 |
| 32 | 1 | 166 | 166 | 166 | 166 | 166 | 166 | 166 | 166 | 166 | 166 | 166 |
| 33 | 0 | 125 | 125 | 125 | 125 | 125 | 125 | 125 | 125 | 125 | 125 | 125 |
| 34 | 0 | 309 | 309 | 309 | 309 | 309 | 309 | 309 | 309 | 309 | 309 | 309 |
| 35 | 0 | 204 | 204 | 204 | 204 | 204 | 204 | 204 | 204 | 204 | 204 | 204 |
| 36 | 0 | 126 | 126 | 126 | 126 | 126 | 126 | 126 | 126 | 126 | 126 | 126 |
| 37 | 0 | 171 | 171 | 171 | 171 | 171 | 171 | 171 | 171 | 171 | 171 | 171 |
| 38 | 0 | 105 | 105 | 105 | 105 | 105 | 105 | 105 | 105 | 105 | 105 | 105 |
| 39 | 0 | 96 | 96 | 96 | 96 | 96 | 96 | 96 | 96 | 96 | 96 | 96 |
| 40* | 0 | 138 | 138 | 138 | 132 | 132 | 150 | 150 | 138 | 150 | 147 | 135 |
| 41* |  | 15 | 12 | 21 | 15 | 18 | - | - | 24 | - | - | 12 |
| The exon structures of carp and human *MYH*s were cited from Muramatsu-Uno et al. [6] and Schachat and Briggs [36], respectively. Coding exons of torafugu were predicted by manual inspection from the genomic sequence of scaffold_139. Exon sizes were identical with four exceptions (for exons 3, 7, 16-17 and 40-41; shown by asterisks). | | | | | | | | | | | | |
|  |  |  |  |  |  |  |  |  |  |  |  |  |
|  |  |  |  |  |  |  |  |  |  |  |  |  |
|  |  |  |  |  |  |  |  |  |  |  |  |  |
|  |  |  |  |  |  |  |  |  |  |  |  |  |
