# Supplementary material for: Lampreys Have a Single Gene Cluster for the Fast Skeletal Myosin Heavy Chain Gene Family
Source: PLoS One. 2013 Dec 20;8(12):e85500. doi: 10.1371/journal.pone.0085500 (PMC3869912; doi:10.1371/journal.pone.0085500)
Supplement: Table S4 — Quantification of GFP or DsRed expressing zebrafish embryos. (DOCX) [file pone.0085500.s004.docx]

| Table S4. Quantification of GFP or DsRed expressing zebrafish embryos | | | |
| --- | --- | --- | --- |
| Construct | Injected | Survival  (% of injected) | Expression in muscle  (% of survival) |
| MYH1-2kb-hrGFP | 123 | 81 (66%) | 20 (25%) |
| MYH1-3kb-hrGFP | 281 | 172 (61%) | 41 (24%) |
| MYH1-5kb-hrGFP | 157 | 86 (55%) | 17 (20%) |
|  |  |  |  |
| MYH2-2kb-DsRed | 133 | 79 (59%) | 17 (22%) |
| MYH2-3kb-DsRed | 263 | 147 (56%) | 30 (20%) |
| MYH2-5kb-DsRed | 125 | 83 (66%) | 19 (23%) |
|  |  |  |  |
| MYH5-2kb-hrGFP | 120 | 78 (65%) | 0 (0%) |
| MYH5-3kb-hrGFP | 132 | 85 (64%) | 0 (0%) |
| MYH5-5kb-hrGFP | 112 | 68 (61%) | 0 (0%) |
