# Supplementary material for: Lampreys Have a Single Gene Cluster for the Fast Skeletal Myosin Heavy Chain Gene Family
Source: PLoS One. 2013 Dec 20;8(12):e85500. doi: 10.1371/journal.pone.0085500 (PMC3869912; doi:10.1371/journal.pone.0085500)
Supplement: Table S5 — Conserved non-coding elements (CNEs) identified by Shuffle-LAGAN. (DOCX) [file pone.0085500.s005.docx]

| Table S5. Conserved non-coding elements (CNEs) identified by Shuffle-LAGAN | | |
| --- | --- | --- |
|  |  |  |
| Table S5A. Lamprey MYH1/MYH2 CNEs identified by Shuffle-LAGAN | | |
| Element | Region | Conserved putative transcription factor binding sites |
| CNE1 | lamprey MYH1: 657-683 | **FOXD3**, **HNF-3beta**, **HFH-3** |
|  | lamprey MYH2: 721-747 |  |
| CNE2 | lamprey MYH1: 735-779 | Oct-1/POU2F1, COMP1 |
|  | lamprey MYH2: 832-878 |  |
| CNE3 | lamprey MYH1: 1858-1888 | Evi-1 |
|  | lamprey MYH2: 2069-2099 |  |
| CNE4 | lamprey MYH1: 1905-1937 | Oct-1/POU2F1, CUTL1 |
|  | lamprey MYH2: 2149-2179 |  |
| CNE5 | lamprey MYH1: 2065-2109 | none |
|  | lamprey MYH2: 2375-2420 |  |
| CNE6 | lamprey MYH1: 2146-2165 | **Oct-1/POU2F1**, CUTL1 |
|  | lamprey MYH2: 2464-2482 |  |
| CNE7 | lamprey MYH1: 2367-2389 | **Oct-1/POU2F1**, Freac-7 |
|  | lamprey MYH2: 2689-2711 |  |
| The putative transcription factor binding site with high probability score is shown in bold (core similarity >0.9, matrix similarity >0.85). | | |
| Table S5B. Lamprey MYH1/zebrafish myhz2 CNEs identified by Shuffle-LAGAN | | |
| Element | Region | Conserved putative transcription factor binding sites |
| CNE1 | lamprey MYH1: 174-193 | none |
|  | zebrafish myhz2: 264-284 |  |
| CNE2 | lamprey MYH1: 607-644 | FOXD3, Freac-7 |
|  | zebrafish myhz2: 705-740 |  |
| CNE3 | lamprey MYH1: 877-902 | Oct-1/POU2F1 |
|  | zebrafish myhz2: 974-996 |  |
| CNE4 | lamprey MYH1: 1510-1547 | **USF** |
|  | zebrafish myhz2: 1595-1636 |  |
| CNE5 | lamprey MYH1: 1621-1651 | none |
|  | zebrafish myhz2: 1722-1755 |  |
| CNE6 | lamprey MYH1: 2063-2086 | FOXD3, Oct-1/POU2F1 |
|  | zebrafish myhz2: 2175-2198 |  |
| CNE7 | lamprey MYH1: 2193-2217 | FOXD3, HNF-3beta, HFH-4 |
|  | zebrafish myhz2: 2288-2315 |  |
| CNE8 | lamprey MYH1: 2329-2360 | HFH-4 |
|  | zebrafish myhz2: 2395-2438 |  |
| CNE9 | lamprey MYH1: 2358-2391 | FOXD3, **Oct-1/POU2F1**, Evi-1 |
|  | zebrafish myhz2: 2436-2468 |  |
| CNE10 | lamprey MYH1: 2722-2742 | **FOXD3**, HNF-3beta, FOXJ2, **SOX-9** |
|  | zebrafish myhz2: 2830-2850 |  |
| CNE11 | lamprey MYH1: 2834-2862 | **FOXD3**, **Nkx2-5** |
|  | zebrafish myhz2: 2937-2966 |  |
| The putative transcription factor binding site with high probability score is shown in bold (core similarity >0.9, matrix similarity >0.85). | | |
|  |  |  |
| Table S5C. Lamprey MYH1/zebrafish myhc4 CNEs identified by Shuffle-LAGAN | | |
| Element | Region | Conserved putative transcription factor binding sites |
| CNE1 | lamprey MYH1: 238-275 | none |
|  | zebrafish myhc4: 158-202 |  |
| CNE2 | lamprey MYH1: 723-749 | Oct-1/POU2F1 |
|  | zebrafish myhc4: 701-724 |  |
| CNE3 | lamprey MYH1: 945-969 | none |
|  | zebrafish myhc4: 924-948 |  |
| CNE4 | lamprey MYH1: 1852-1875 | FOXD3, **Nkx2-5**, FOXJ2 |
|  | zebrafish myhc4: 1708-1731 |  |
| CNE5 | lamprey MYH1: 1913-1942 | CUTL1 |
|  | zebrafish myhc4: 1788-1815 |  |
| CNE6 | lamprey MYH1: 1962-1996 | FOXD3, COMP1 |
|  | zebrafish myhc4: 1866-1900 |  |
| CNE7 | lamprey MYH1: 2146-2173 | **Oct-1/POU2F1**, CUTL1 |
|  | zebrafish myhc4: 2101-2129 |  |
| CNE8 | lamprey MYH1: 2314-2341 | FOXD3, **TATA** |
|  | zebrafish myhc4: 2327-2354 |  |
| CNE9 | lamprey MYH1: 2362-2390 | FOXD3, **HNF-3beta** |
|  | zebrafish myhc4: 2360-2385 |  |
| CNE10 | lamprey MYH1: 2420-2439 | CUTL1 |
|  | zebrafish myhc4: 2443-2462 |  |
| CNE11 | lamprey MYH1: 2603-2622 | none |
|  | zebrafish myhc4: 2635-2653 |  |
| The putative transcription factor binding site with high probability score is shown in bold (core similarity >0.9, matrix similarity >0.85). | | |
|  |  |  |
| Table S5D. Mouse MYH1/zebrafish myhz2 CNEs identified by Shuffle-LAGAN | | |
| Element | Region | Conserved putative transcription factor binding sites |
| CNE1 | mouse MYH1: 844-876 | Oct-1/POU2F1 |
|  | zebrafish myhz2: 1091-1117 |  |
| CNE2 | mouse MYH1: 922-947 | Oct-1/POU2F1 |
|  | zebrafish myhz2: 1152-1181 |  |
| CNE3 | mouse MYH1: 1263-1281 | none |
|  | zebrafish myhz2: 1542-1560 |  |
| CNE4 | mouse MYH1: 1655-1678 | Oct-1/POU2F1, HNF-4 |
|  | zebrafish myhz2: 1913-1941 |  |
|  |  |  |
| Table S5E. Mouse MYH1/zebrafish myhc4 CNEs identified by Shuffle-LAGAN | | |
| Element | Region | Conserved putative transcription factor binding sites |
| CNE1 | mouse MYH1: 420-451 | Oct-1/POU2F1 |
|  | zebrafish myhc4: 571-599 |  |
| CNE2 | mouse MYH1: 643-671 | Oct-1/POU2F1 |
|  | zebrafish myhc4: 861-889 |  |
| CNE3 | mouse MYH1: 739-774 | Pax-4 |
|  | zebrafish myhc4: 1010-1045 |  |
| CNE4 | mouse MYH1: 862-894 | Oct-1/POU2F1, Pax-6 |
|  | zebrafish myhc4: 1128-1163 |  |
| CNE5 | mouse MYH1: 1699-1715 | none |
|  | zebrafish myhc4: 1844-1858 |  |
| CNE6 | mouse MYH1: 1874-1908 | Oct-1/POU2F1 |
|  | zebrafish myhc4: 2002-2036 |  |
| CNE7 | mouse MYH1: 1942-1967 | HNF-1, **TATA** |
|  | zebrafish myhc4: 2087-2114 |  |
| The putative transcription factor binding site with high core and matrix similarity is shown in bold (core similarity >0.9, matrix similarity >0.85). | | |
|  |  |  |
