# Supplementary figures and images for: Lampreys Have a Single Gene Cluster for the Fast Skeletal Myosin Heavy Chain Gene Family
Source: PLoS One. 2013 Dec 20;8(12):e85500. doi: 10.1371/journal.pone.0085500 (PMC3869912; doi:10.1371/journal.pone.0085500)

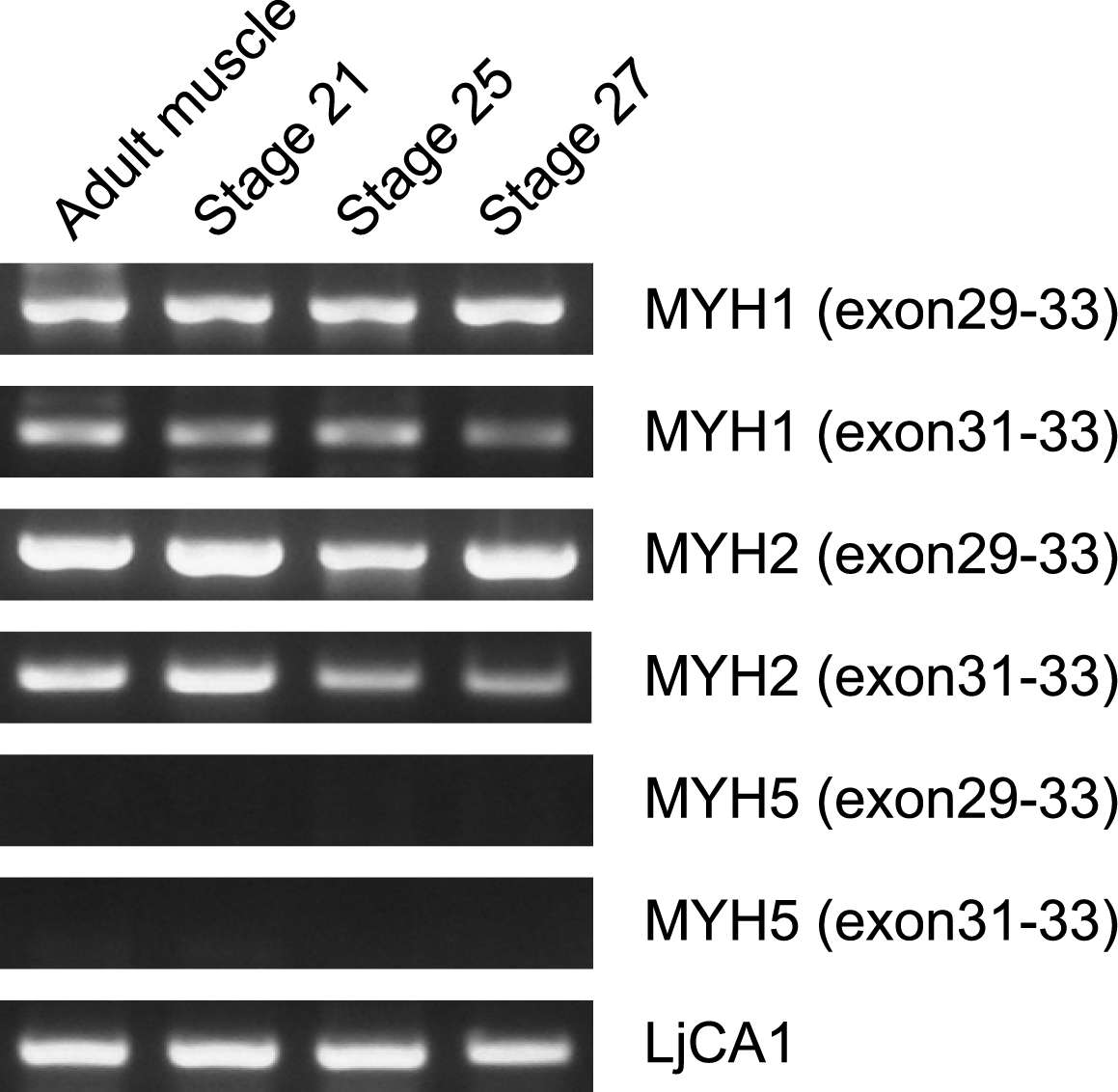

Supplement: Figure S3 — Expression analysis of Japanese lamprey myosin heavy chain genes (MYHs). The target regions for amplification of each MYH are shown in brackets. The gene encoding cytoplasmic actin LjCA1 was used as the positive control. (TIF) [file pone.0085500.s008.tif]

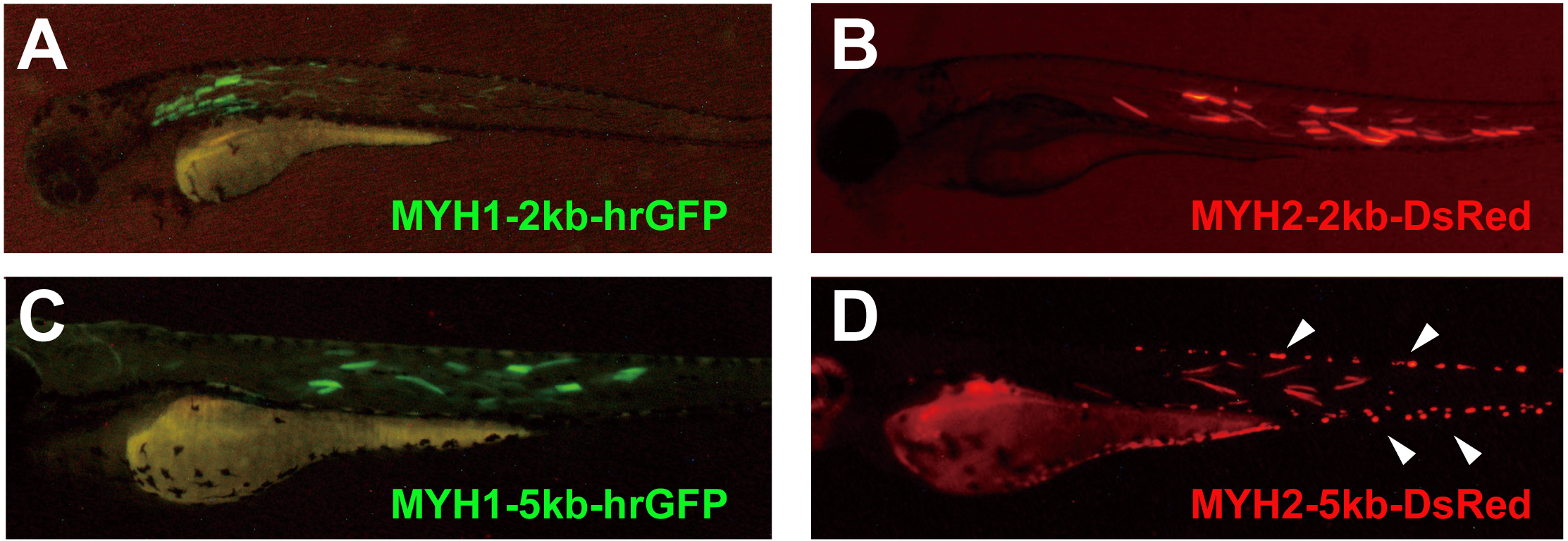

Supplement: Figure S4 — Transient expression of the Japanese lamprey MYH1-2kb-hrGFP, MYH1-5kb-hrGFP, MYH2-2kb-DsRed and MYH2-5kb-DsRed transgenes in zebrafish embryos. Lateral view showing the expression of the MYH1-2kb-hrGFP (A), MYH2-2kb-DsRed (B), MYH1-5kb-hrGFP (C) and MYH2-5kb-DsRed (D) in the trunk myotome of zebrafish embryos at 3 days post-fertilization. Arrowheads indicate autofluorescence due to pigment cells on the embryo surface. (TIF) [file pone.0085500.s009.tif]

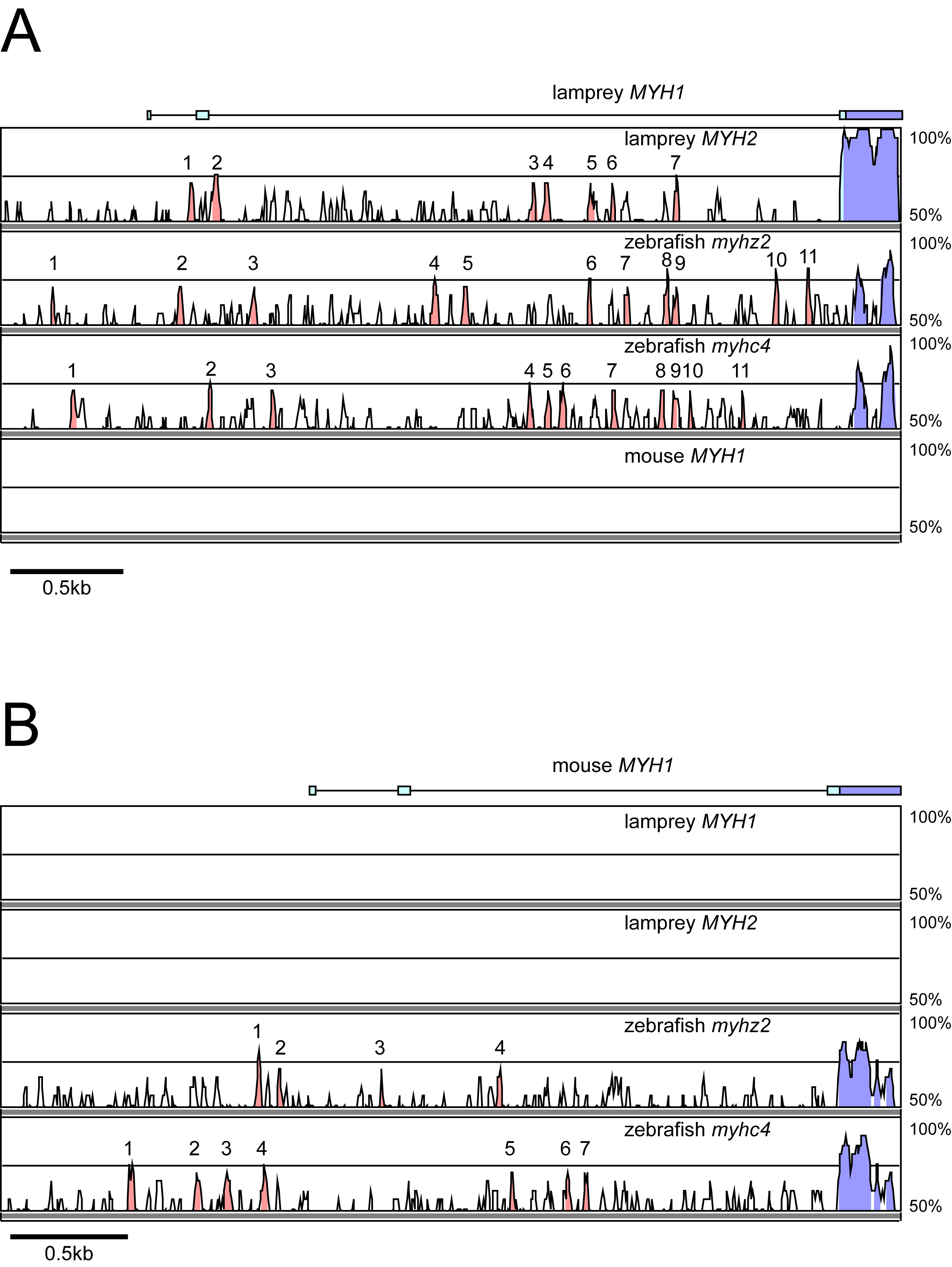

Supplement: Figure S5 — Shuffle-LAGAN alignment of the 5' flanking sequences of myosin heavy chain genes (MYHs). The 5' flanking sequences of Japanese lamprey MYH1 and MYH2, zebrafish myhz2 and myhc4 and mouse MYH1 are aligned with Shuffle-LAGAN and visualized with mVISTA. The 5' flanking sequences of Japanese lamprey MYH1 (A) and mouse MYH1 (B) are used as baselines. Coding (blue) and non-coding (light blue) exons are annotated. Pink peaks represent non-coding conservation above 70% over at least 20 bp. (TIF) [file pone.0085500.s010.tif]
